# Impaired FGF10 Signaling and Epithelial Development in Experimental Lung Hypoplasia With Esophageal Atresia

**DOI:** 10.3389/fped.2018.00109

**Published:** 2018-04-20

**Authors:** Jun Wang, Hao Liu, Linlin Gao, Xiaomei Liu

**Affiliations:** ^1^Key Laboratory of Maternal-Fetal Medicine of Liaoning Province, Department of Obstetrics and Gynecology, Shengjing Hospital of China Medical University, Shenyang, China; ^2^Department of Obstetrics and Gynecology, Benxi Central Hospital of China Medical University, Benxi, China; ^3^Central Laboratory, Shengjing Hospital of China Medical University, Shenyang, China

**Keywords:** esophageal atresia, lung, Fgf10, Ctsh, epigenetic

## Abstract

Patients with esophageal atresia (EA) and tracheoesophageal fistula (TEF) often experience persistent respiratory tract disease. In experimental models, doxorubicin-induced developmental lung abnormalities may result from downregulation of branching morphogenesis factor fibroblast growth factor (Fgf10). This study investigated the temporospatial expression of Fgf10 pathway components and lung epithelial factors in an doxorubicin-induced EA-TEF model by quantitative polymerase chain reaction, immunohistochemistry, and immunoblotting. Epigenetic regulation of gene expression by histone deacetylation was also investigated. Bone morphogenetic protein (Bmp) 4 and Cathepsin H (Ctsh), downstream targets of *Fgf10*, were significantly downregulated in the EA-TEF model during the saccular stage, consistent with Fgf10 expression. The developmental expression pattern of P2x7 receptor (ATI-cell marker), *Sftpa*, and *Sftpb* in lung epithelial cells was not affected. *Sftpc* (ATII-cell Marker) and *Scgb1a1* (Clara cell marker) were significantly downregulated at the canalicular stage. Meanwhile, histone deacetylase (Hdac) 1 was upregulated and subsequently decreased acetylation of histone H3 Lys56 in the EA-TEF model, which returned to a normal level at the saccular stage. In conclusion, disturbed molecular signaling involving Fgf10/Ctsh was associated with impaired airway branching and epithelial cell development in lung morphogenesis, as evidenced by downregulated Sftpc and Scgb1a1 protein expression. The influence of Hdac1 activity on gene and protein expression in lung epithelial cells deserves further study.

## Introduction

Esophageal atresia (EA) is the most frequent congenital anomaly of the esophagus, occurring in approximately one of every 4099 births worldwide [1]. Abnormal separation of the foregut into the esophagus and trachea during development results in lack of esophageal continuity with or without an abnormal connection of the esophagus and trachea and formation of a tracheo-esophageal fistula (TEF). Neonates with EA-TEF may have respiratory malformations, which are found in 2.8% of patients and in 13.2% of autopsies [2]. EA-TEF survivors often experience persistent nonspecific respiratory tract disease with chronic cough, asthma-like symptoms, recurrent pneumonia or bronchiectasis, and chronic bronchitis [3] that do not improve or become more frequent with age [4, 5]. Gastro-esophageal reflux, peptic bronchitis, post-thoracotomy chest wall deformities, or the fistula stump account for these long-term sequelae. Defects in the airway or lung parenchyma or lung hypoplasia may also be involved [6–8].

Rat lung development includes embryonic, pseudoglandular, canalicular, saccular, and alveolar stages. It is a highly regulated process directed by mesenchymal-epithelial interactions, which coordinate the spatiotemporal expression of multiple regulatory factors [9]. Due to the limited availability of lung tissue sample from human subjects, rodent model was applied to perform basic research. Doxorubicin (Adriamycin)-exposed fetal rats, has been widely used because of closely mimics the human phenotype, with up to 70% of tracheo-esophageal malformations [10, 11]. Deficient embryonal lung branching and lung hypoplasia in fetal rats with EA-TEF caused by doxorubicin exposure is accompanied by downregulated fibroblast growth factor 10 (Fgf10) at the saccular stage (E21) [10]. During lung development, signaling by Fgf10 and its epithelial tyrosine kinase transmembrane receptor, fibroblast growth factor receptor 2b (Fgfr2b), are required for coordinated induction of several genes that control proliferation, differentiation, and branching of the lung epithelial tubules [12]. Lungs formation does not occur in genetically modified mice in which Fgf10 or its receptor Fgfr2b have been deleted [13, 14]. Activation of Fgf10-Fgfr2b signaling has induces the expression of bone morphogenetic proteins in lung epithelium [15]. The study was designed to determine whether disruption of Fgf10 signaling induces defective branching morphogenesis in EA-TEF. The study objective was to assay spatiotemporal expression of Fgf10 signaling pathway participants and lung epithelial markers in a rat model of EA-TEF caused by intrauterine exposure to doxorubicin. The underlying epigenetic mechanism of changes of gene expression was also investigated.

## Materials and methods

### Animals

This study was carried out in accordance with the National Institutes of Health (NIH) Guide for the Care and Use of Laboratory Animals recommended by Animal Research Committee of China Medical University. The protocol was approved by the Animal Research Committee of China Medical University. Rats weighing 230–260 g (Changsheng Biotechnology, Benxi, China) were housed individually at 21–22°C on a 12:12 h light-dark cycle. Timed-pregnant Wistar rats were bred in house, and the day that a vaginal plug was found was day 0. Doxorubicin -treated rats received three intraperitoneal injections of 1.75 mg/kg doxorubicin (Haizheng Pharmaceutical Co., Taizhou, China) in saline on embryonic days E7, E8, and E9 as previously described [10]. Control rats received an equivalent volume of saline on the same schedule. A subset of dams was killed by injection of potassium chloride on E15, E18, and E21. The chests of the fetus were dissected and whose trachea which was directly connected to the lung and stomach was considered to be esophageal atresia (Figure S1). The embryonic lungs were removed and dissected free from the trachea.

### Morphometry

Immunohistochemical (IHC) staining of fixed lung tissue was performed as previously described [10]. After tracheal cannulation, 4% paraformaldehyde was injected at <10 cm/H_2_O for alveolar distention, the fetal lungs were collected, fixed in formalin, embedded in paraffin, and sectioned. Tissue sections were stained with hematoxylin and eosin for evaluation of general tissue morphology. IHC staining was performed on 3 μm serial sections using standard techniques. Briefly, after rehydration in a graded alcohol series, sections were boiled in 0.01 M sodium citrate for antigen retrieval prior to antibody incubation. Sections were incubated overnight with primary antibodies including rabbit polyclonal anti-Scgb1a1 (1:200, 26909-1-AP, Proteintech), anti-Pla2g4a (1:100, 10827-1-AP, Proteintech) and mouse monoclonal anti-histone deacetylase (Hdac1, 1:200, 10197-1-AP, Proteintech). Negative controls were included. Imaging at low (200×) and high (400×) magnification was taken on a Nikon ECLIPSE Ti microscope. All images were analyzed with NIS-Elements BR 2.10 image software (Nikon, Tokyo, Japan).

#### RNA extraction and quantitative PCR

Expression of *Fgf10* signal-related factors, lung proximal and distal epithelium markers, and epigenetic regulation factors in fetal lung were assayed by quantitative PCR. Total RNA was extracted from snap-frozen lungs by Trizol reagent (Invitrogen, Carlsbad, CA) following the manufacture's protocol, and the concentration was determined spectrophotometrically in duplicate (Nano Vue; GE Healthcare, Buckinghamshire, England). Reverse transcription and amplification were performed as previously described [16]. Amplification was performed with a LightCycler 480 SYBR Green I Master (Roche, Mannheim, Germany) in 20 μL of reaction solution with specific primers for target genes. Quantitative PCR was performed in multiple cycles using a 7500 Fast thermal cycler (ABI, Inc., USA) with denaturation at 94°C for 1min, annealing at 60°C for 50s, and elongation at 72°C for 0.5min. The specific primers and annealing temperature of each gene are listed in Table 1. The specificity of the PCR products was confirmed by analysis of the dissociation curve. The expected amplicon size, and the absence of nonspecific products were confirmed by analysis of the PCR products on 2% agarose gels, followed by ethidium bromide staining and visualization under UV light. The relative mRNA levels were calculated using the 2^−ΔΔCt^ method after normalization against β*-actin* as a housekeeping gene.

**Table 1:** Primers used in quantitative real-time PCR.

**Gene name**	**Accession number**	**Primer sequences**	**Size (bp)**
*Shh*	NM_017221	F: 5- AAAGCTGACCCCTTTAGCCTA-3	103
		R: 5- TTCGGAGTTTCTTGTGATCTTCC-3	
*Fgfr2b*	NM_001109896	F: 5- ACCCGACAAGAGAACGCTTACCAT-3	92
		R: 5-TGTCACATGACGGTAGCAAGGTGA-3	
*Ctsh*	NM_012939	F: 5- TTGGCTATGGAGAACAGAA-3	96
		R: 5- CACGCTCAATGAGGAAGTA-3	
*Bmp4*	BC078901	F: CAGAGCCAACACTGTGAGGA-3	108
		R: GGGATGCTGCTGAGGTTAAA-3	
*Bmpr1a*	S75359	F: 5- GCCACCCTGGACACCAGAGC-3	101
		R: 5- GCAGGCTTGCCTTGCGTG-3	
*Bax*	U49729	F: 5- AAACTGGTGCTCAAGGCCCT-3	92
		R: 5- AGCAGCCGCTCACGGAG-3	
*Bcl-2*	NM_016933.1	F: 5-CCGGGAGAACAGGGTATGATAA-3	81
		R: 5- CCCACTCGTAGCCCCTCTG-3	
*Bim*	NM_022612	F: 5- GAGAAGGTGGACAATTGCAGC-3	91
		R: 5- CTCCTGTCTTGCGATTCTGTCT-3	
*Sftpa*	NM_001270645	F: 5- TACCAGAGCAGGAGGCAACA-3	68
		R: 5- CAATACTTGCAATGGCCTCGTT-3	
*Sftpb*	NM_138842.1	F: 5- CCATCCCTCTGCCCTTCTG-3	73
		R: 5- CACCCTTGGGAATCACAGCTT-3	
*Sftpc*	NM_017342.2	F: 5-TCCCAGGAGCCAGTTTCG-3	61
		R: 5- CACGATGAGAAGGCGTTTGA-3	
*P2x7r*	NM_019256.1	F: 5- CATGGAAAAGCGGACATTGA-3	66
		R: 5- CCAGTGCCAAAAACCAGGAT-3	
*Scgb1a1*	NM_013051.1	F: 5- CGGACATCTGCCCAGGATTTCT-3	208
		R: 5- ACACAGAGGACTTGTTAGGAT-3	
*Pla2g4a*	NM_133551.2	F: 5-GAAGTTTGCTCATGCCCAGACCT-3	234
		R: 5- TTCATAGAGCGCCTTCATCACACC-3	
*Pla2g2a*	NM_031598.3	F: 5- CCCCAAGGATGCCACAGATT-3	201
		R: 5-TTCCGGGCAAAACATTCAGC-3	
*DNM_t-1*	NM_013062	F: 5- AACGGAACACTCTCTCTCACTCA-3	148
		R: 5- TCACTGTCCGACTTGCTCCTC-3	
*Hdac-1*	NM_001025409	F: 5- CCTCACCGAATCCGAATG-3	145
		R: 5- CGAATAGAACGCAAGAACTTG-3	
*Hdac-2*	NM_053447	F: 5- TCAAGTTTCTACGATCAATAAGG-3	77
		R: 5- CTTCTCCGACATTAAATCTCTG-3	
*Hdac-5*	NM_053450	F: 5-AGCACCGAGGTAAAGCTGAG-3	104
		R: 5- GCTGTGGGAGGGAATGGTT-3	
*β-actin*	NM_031144.3	F: 5- AGTCCCTCACCCTCCCAAAAG-3	97
		R: 5- AAGCAATGCTGTCACCTTCCC-3	

*β-actin was used as the housekeeping gene*.

#### Immunoblotting

Immunoblotting was used to assay target protein expression in the rat embryo lungs. Total protein was extracted from frozen lung tissue with RIPA lysis buffer following to the kit manufacturer's (P0013B, Beyotime, China) instructions. Histones were extracted from lung tissues using EpiQuik total histone extraction kits (EpiGentek Group Inc., Farmingdale, NY, USA). The protein concentration was measured with a bicinchoninic acid assay kit (Pierce Biotechnology, Waltham, MA, USA). Equal amounts of total protein were separated by electrophoresis on 6–12% sodium dodecyl sulfate polyacrylamide gel electrophoresis, depending on the molecular weights of the target proteins, and transferred to polyvinylidene fluoride(PVDF) membranes (Millipore, MA, USA) as described by Towbin et al. [17]. After blocking the PVDF membrane with 5% nonfat milk in Tween-20 containing Tris buffered saline for 2h at room temperature, the blots were immunoprobed with rabbit polyclonal anti-Ctsh (1:400, Proteintech, China), anti-Hdac1 (1:800, Proteintech) and mouse monoclonal anti-β-actin (1:5,000, Proteintech) overnight at 4°C. The membranes were probed with a horseradish peroxidase-conjugated secondary antibody for 2h at room temperature. The antigen-antibody complexes were visualized with an electrochemiluminescence (ECL) detection kit (Thermo Scientific, USA). The optical densities of the target proteins in each sample was measured with a gel reader and normalized to β-actin expression. The expression of H3K56ac (A7256, ABclonal, Inc., Woburn, MA, USA) was determined by immunoblotting with H3 (E1A6359; EnoGene Biotech Co. Ltd., Nanjing, China) as the loading control. Assays were performed in triplicate.

#### Statistical analysis

Data were reported as means ± SEM and the probability value used for statistical significance was *P* < 0.05. Statistical analysis was performed with GraphPad Software (San Diego, CA, USA). Between-group differences in nonparametric data were analyzed by the Mann–Whitney test for two groups and the Kruskall–Wallis test for more than two groups. Differences in continuous data were tested by parametric methods, ANOVA or Student's *t*-test.

## Results

### Lung branching-associated factors in the hypoplastic lungs

**Figure 1:**
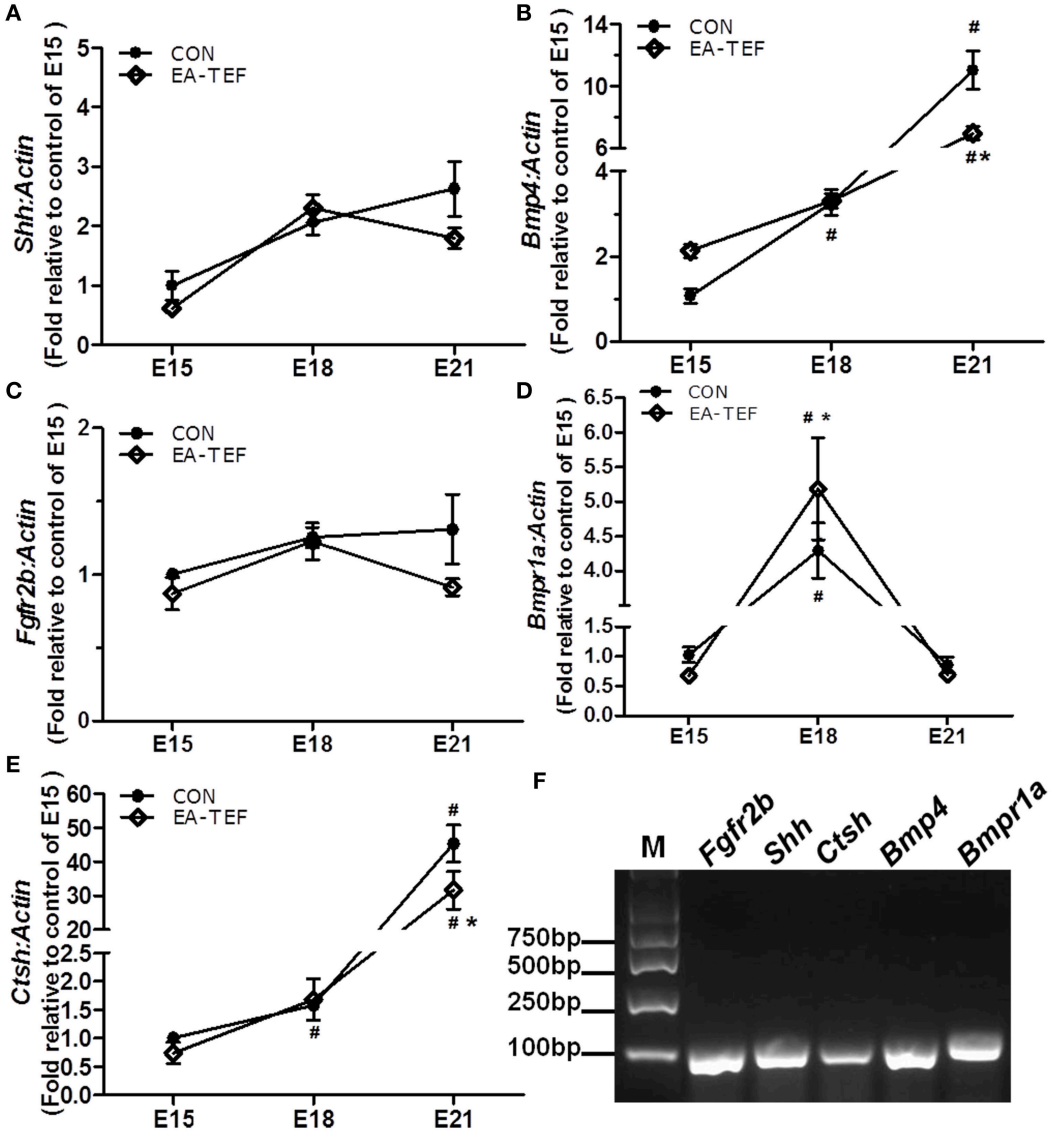
Kinetics of *Fgf10* signaling pathway factors in fetal lungs. **(A–E)** Shh, Fgfr2b, Bmp4, Bmpr1a, and Shh mRNA expression was assayed by quantitative PCR in EA and control lungs during the pseudoglandular (E15), canalicular (E18), and saccular (E21) stages, and results are shown relative to controls at E15. Data are means ± SEM, ^*^P < 0.05, EA-TEF vs. age-matched controls between group; ^#^*P* < 0.05, E18 or E21 vs. E15 within group (each *n* = 6). Due to the huge difference between the values in the same chart, the error bars of some of the points are too small to display. The data of *Shh*- EA-TEF-E15 is 0.618 ± 0.031; the data of *Ffgr2b*- CON-E15 is 1.002 ± 0.032; the data of *Bmpr1a*- EA-TEF-E15 is 0.679 ± 0.059. **(F)** Representative 2% agarose gel electrophoresis images of the amplicon size and the specificity of PCR products.

**Figure 2:**
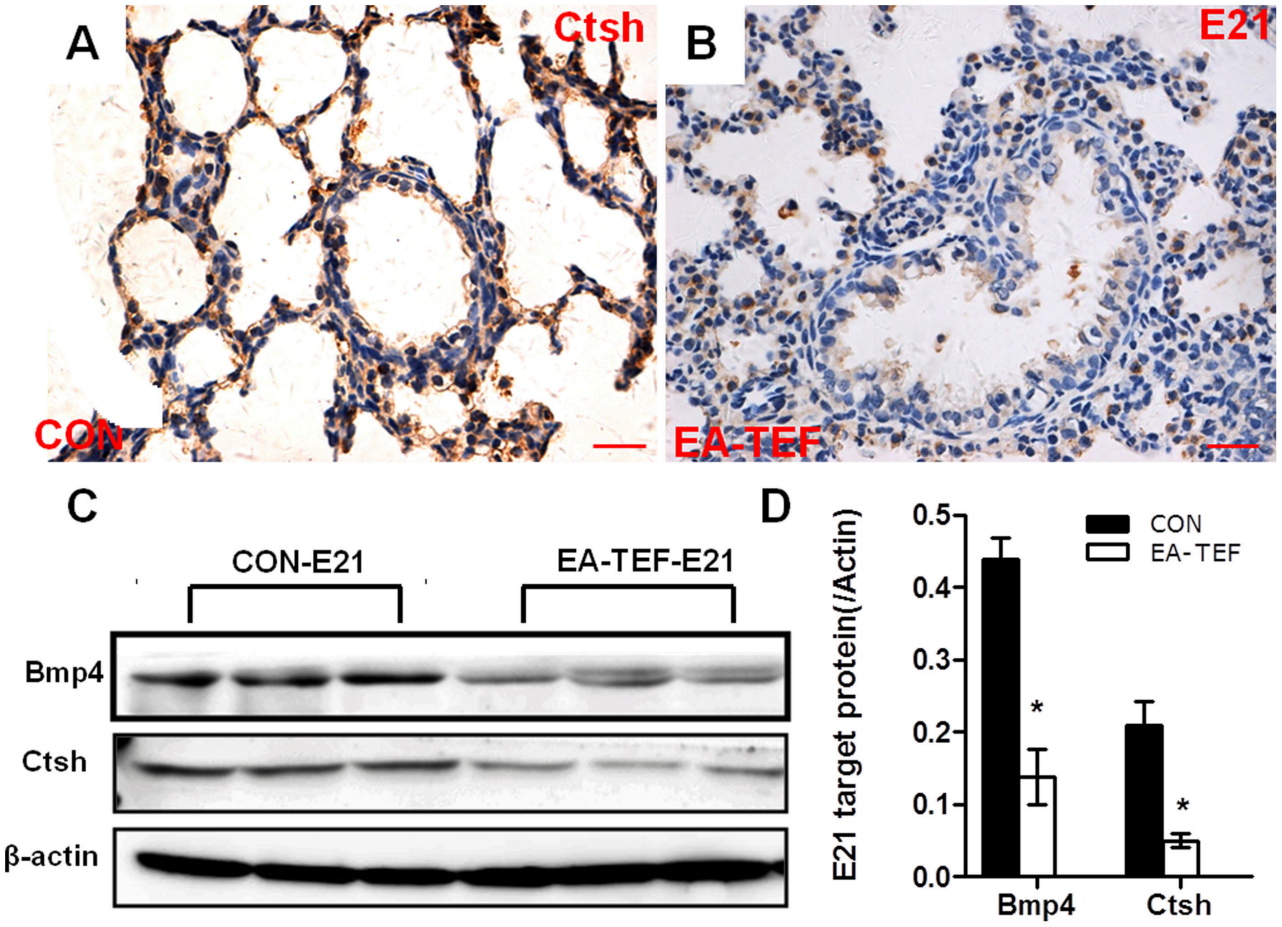
Histological localization and quantification of Ctsh and Bmp4 protein in fetal lungs. **(A,B)** Representative photomicrographs of IHC- staining for Ctsh in the lung sections from Con **(A)** and EA-TEF **(B)** of E21. (Original magnification ×400, scale bar = 100 μm). Ctsh was mainly expressed in the bronchial epithelial cells. Ctsh protein seemed decreased in EA lungs compared with age-matched control. **(C,D)** Representative immunoblotting and densitometric analysis of Bmp4 and Ctsh protein expression in fetal lungs at E21 normalized against β-actin expression. ^*^P < 0.05, vs. control at the same time point.

We previously reported that primary lung maldevelopment caused by deficient airway branching in experimental EA-TEF, and *Fgf10*, a key mesenchymal regulator of the process of endodermal branching was significantly deficient on E21 [10]. Expression of *Fgf10* pathway components in hypoplastic lungs including its specific cell surface receptor (*Fgfr2b*), negative regulator sonic hedgehog (*Shh*), and the downstream targets *Bmp4* and *Ctsh* was assayed. The expression of Shh, an epithelial gene known to negatively regulate Fgf10 expression, was unchanged compared with that in control lungs (Figure 1A), indicating that other distal epithelial signals regulate mesenchymal Fgf10 expression. No significant differences were observed in the gene expression pattern of receptor *Fgfr2b* and *Bmpr1a*, while *Bmp4* expression tended to be lower at E21 in hypoplastic lung tissue (Figures 1B–D). In the saccular stage, *Ctsh* mRNA was significantly decreased in hypoplastic lungs compared with the controls (Figure 1E), similar to the expression of *Fgf10*. The specificity of primers for Fgf10 pathway factors was confirmed by agarose gels electrophoresis (Figure 1F). The differences in Bmp4 and Ctsh protein expression indicated by immunoblotting and IHC were consistent with the quantitative PCR results (Figures 2A–D).

### Lung epithelium-associated factors in hypoplastic lungs

**Figure 3:**
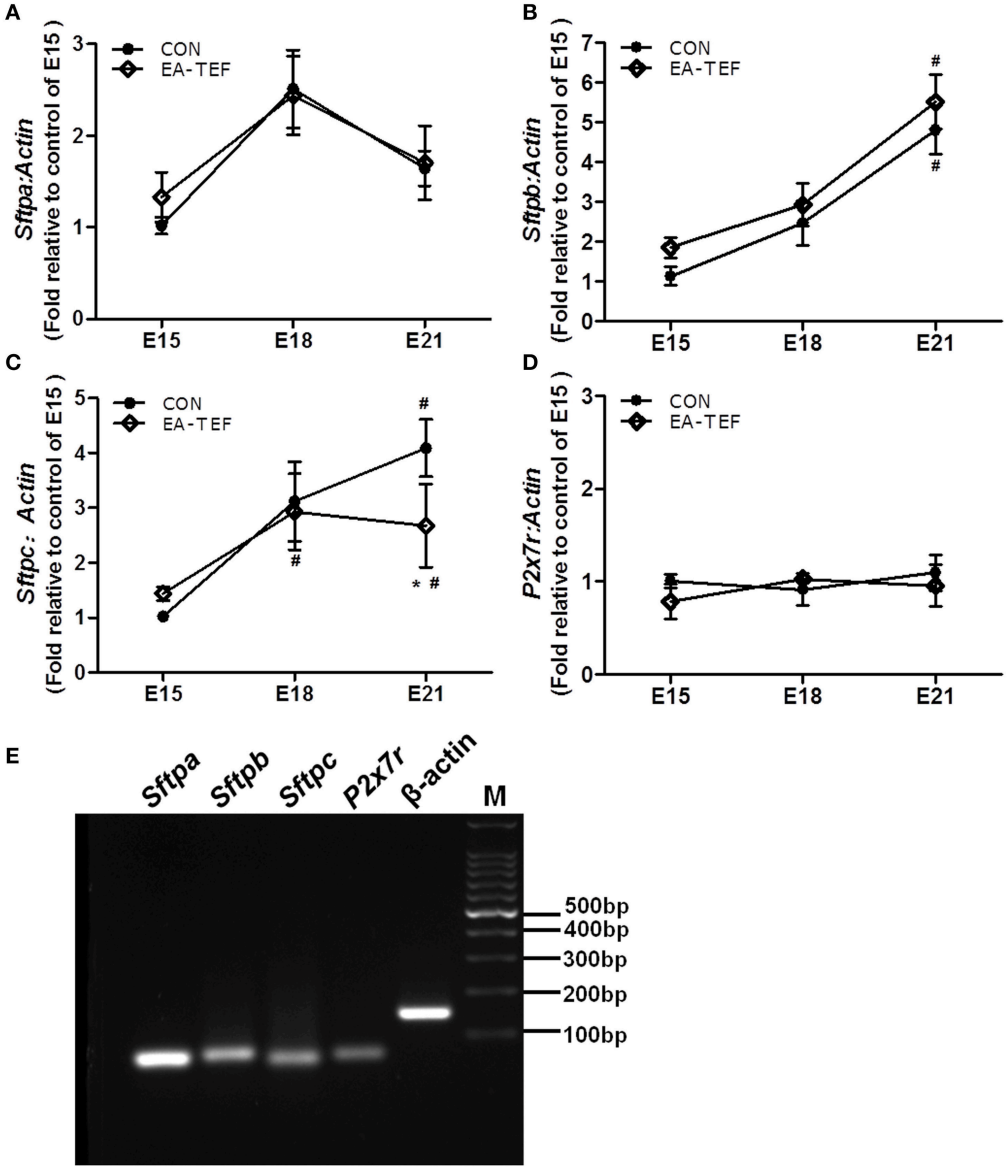
Effect of doxorubicin on expression of epithelial markers in fetal lungs. **(A–D)** *P2x7r, Sftpa, Sftpb*, and *Sftpc* mRNA expression in both groups was assayed by quantitative PCR, and **(E)** the specificities of the PCR products were confirmed in 2% agarose gels. Expression is relative to an E15 control and normalized against a β*-actin* housekeeping gene. Data are means ± SEM of five rats, ^*^P < 0.05, EA-TEF vs. age-matched controls, between group; ^#^*P* < 0.05, E18 or E21 vs. E15, within group. In **(C)**, the data of *Sftpc*-control -E15 is (1.019 ± 0.092), the corresponding error bars is too small to display.

**Figure 4:**
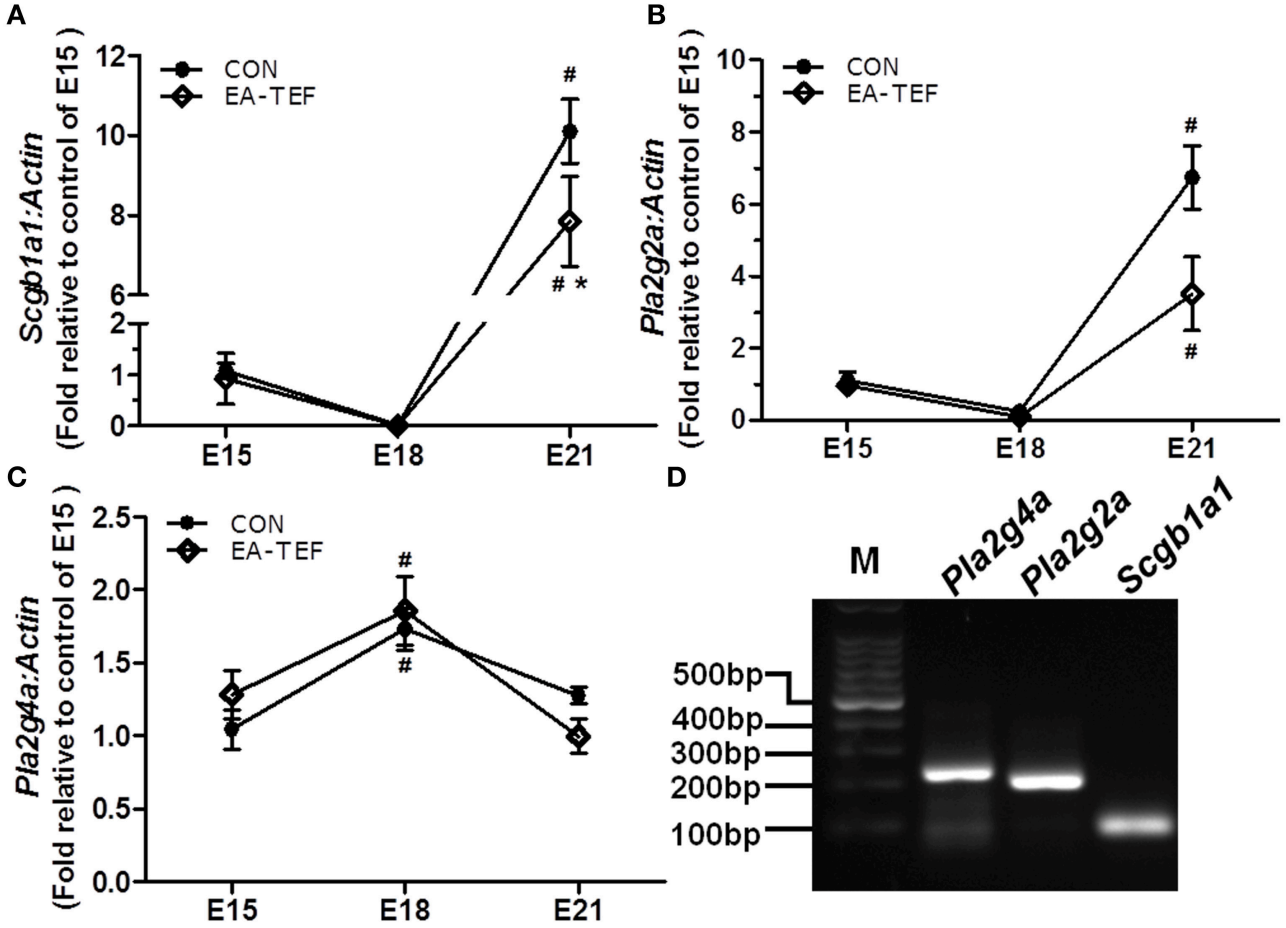
Effect of doxorubicin on *Scgb1a1* and *Pla2* expression in fetal lungs. **(A–C)** Expression of *Scgb1a1* and its downstream targets *Pla2g4a* and *Pla2g2a* mRNAs in the fetal lungs of both groups were assayed by quantitative PCR and **(D)** the specificity of the PCR products was confirmed visually on 2% agarose gels. Expression is reported relative to E15 controls and normalized against a β*-actin* housekeeping gene. Data are means ± SEM of five rats, ^*^P < 0.05, EA-TEF vs. age-matched controls, inter-group; ^#^*P* < 0.05, E18 or E21 vs. E15, within group.

**Figure 5:**
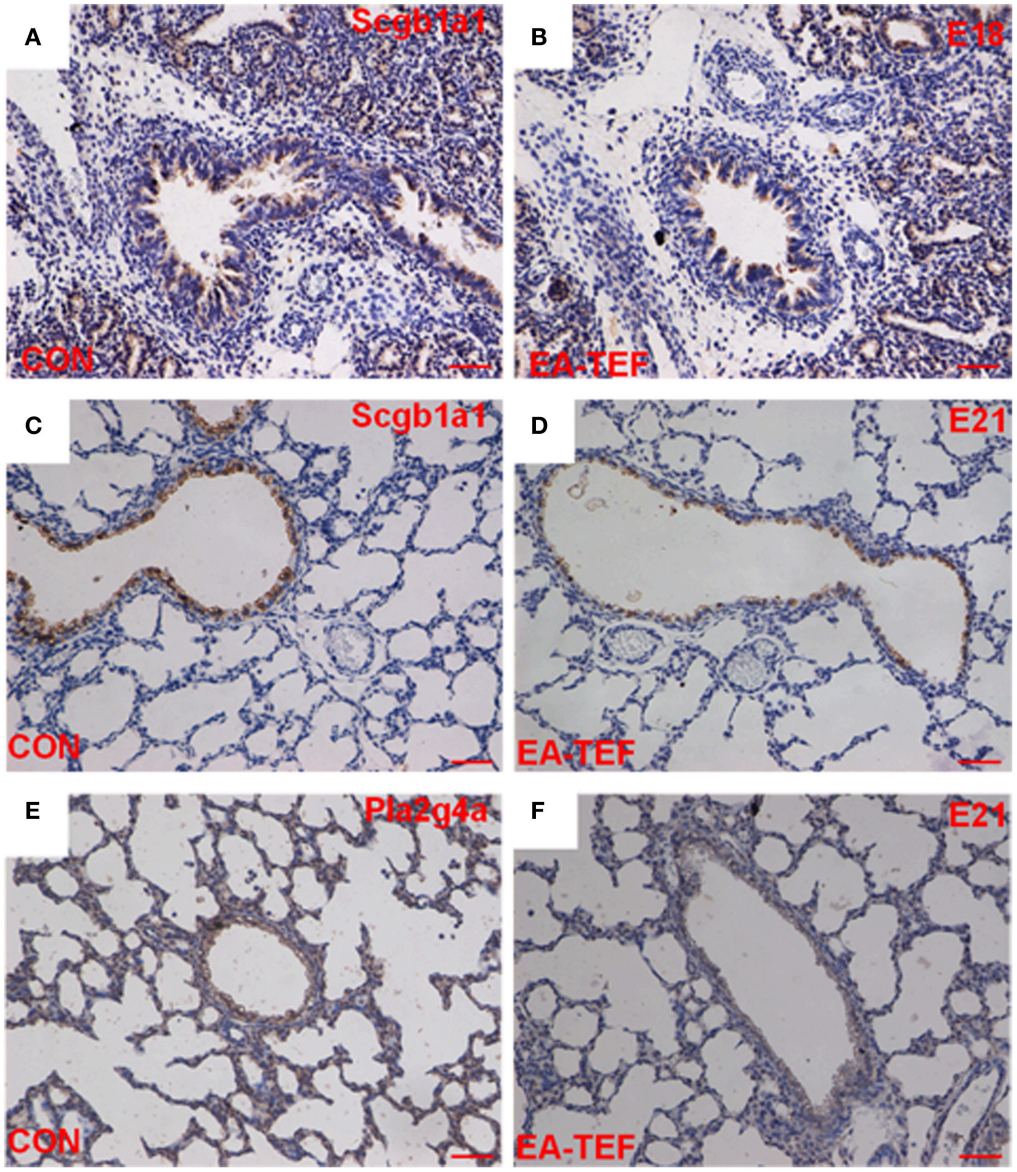
Histological localization and quantification of Scgb1a1 and Pla2 protein in fetal lungs. **(A–D)** Representative photomicrographs of IHC- staining for Scgb1a1 in sections of lung tissue from control **(A,C)** and EA-TEF **(B,D)** embryos at E18 (upper panel) and E21 (lower panel). (Original magnification ×200, scale bar = 50 μm). Scgb1a1 was primarily expressed in bronchial epithelial cells. At E21, Scgb1a1 staining intensity decreased in EA lungs compared with age-matched controls. **(E,F)** Representative IHC staining of Pla2g4a in the lung tissue sections from Control **(E)** and EA-TEF **(F)** embryos at E21. No changes were found in the expression and localization pattern of Pla2g4a.

To investigate the impairment of the development of fetal lung epithelium by doxorubicin, we determined the expression of genes coding for epithelial cell proteins including purinergic ligand-gated ion channel 7 receptor (P2X7R), an alveolar epithelial type I (ATI) cell marker, surfactant protein (SP)A, Sftpb, Sftpc, alveolar epithelial type II (ATII) markers, and Clara cell secretary protein (SCGB1A1), a, Clara cell marker. Quantitative PCR found that, as gestational age increases, the developmental patterns of *P2x7r, Sftpa*, and *Sftpb*, mRNA expression in normal and hypoplastic lungs were similar. *P2x7r* and *Sftpa* expression were relatively constant, and *Sftpb* expression increased from E15 to E18 (Figures 3A–D). In contrast, at E21 *Sftpc* mRNA level was lower in hypoplastic lungs than in control lungs (Figure 3C). In both group, *Scgb1a1* mRNA decreased slightly at E18, and then increased significantly at E21. Like Sftpc, *Scgb1a1* expression was downregulated in hypoplastic, compared with control lungs at the saccular stage (Figure 4A). The specificity of primers for lung epithelium-associated factors was confirmed by agarose gels electrophoresis (Figures 3E, 4D). Assay of the spatial and temporal expression of Scgb1a1 protein by IHC found that it was mainly expressed in bronchial epithelial cells. No differences in the localization pattern of Scgb1a1 protein in normal and hypoplastic lungs were observed in the canalicular and saccular stages. At E21, the IHC staining intensity of Scgb1a1 protein decreased in embryos with EA compared with age-matched control (Figures 5A–D). The expression of phospholipase A2 (including intracellular *Pla2g4a* and secreted *Pla2g2a*), a key enzyme in pulmonary inflammation and cell injury that is inhibited by Scgb1a1 [18] was unchanged (Figures 4B,C). The spatial expression pattern of Pla2g4a protein was also unchanged between two groups (Figures 5E,F).

### Epigenetic regulatory factors in hypoplastic lungs

**Figure 6:**
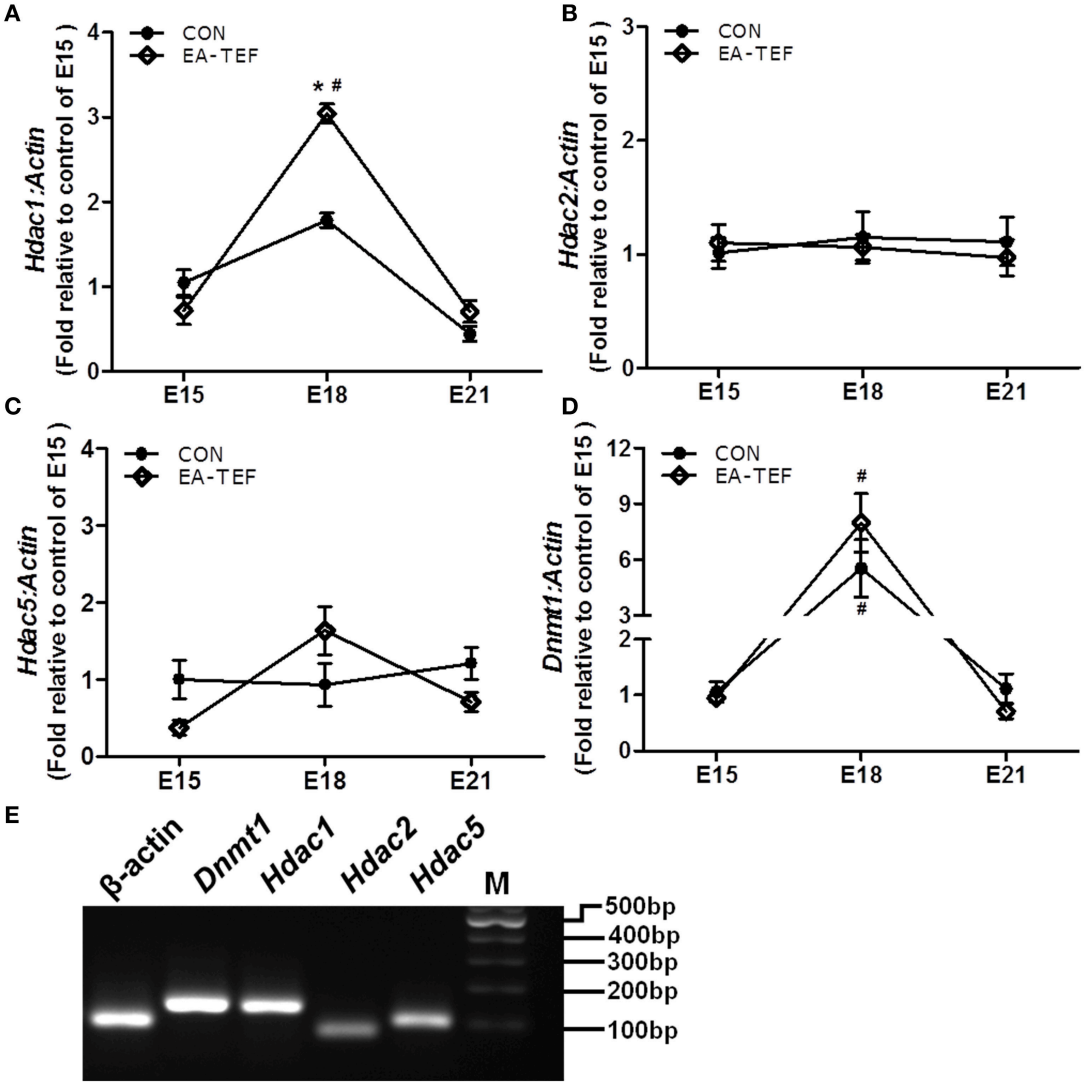
Effect of doxorubicin on expression epigenetic regulatory factors in fetal lungs. **(A–D)** *Dnmt1*, *Hdac1, Hdac2*, and *Hdac5* mRNA expression were determined by quantitative PCR relative to E15. **(E)** the specificity of the PCR products was confirmed visually on 2% agarose gels. Data are means ± SEM, ^*^P < 0.05, EA-TEF vs. age-matched controls, between group; ^#^*P* < 0.05, E18 or E21 vs. E15, within group.

**Figure 7:**
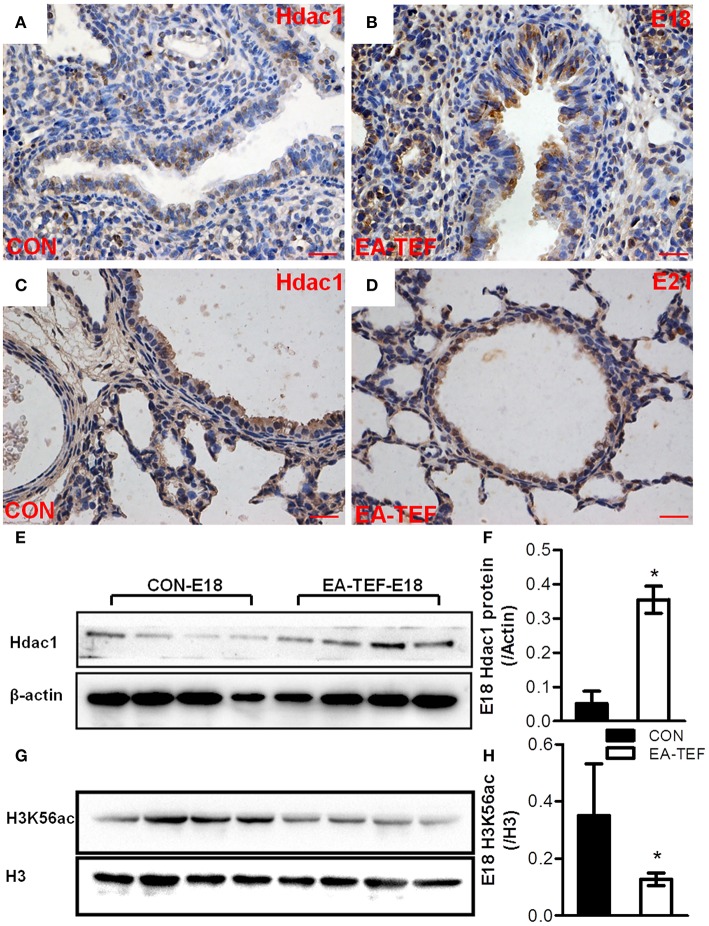
Hdac1 protein expression and localization in fetal lungs. **(A–D)** Representative photomicrographs of IHC staining for Hdac1 in lung tissue sections from Control **(A,C)** and EA-TEF **(B,D)** embryos of E18 (upper panel) and E21 (lower panel). Hdac1 staining is seen throughout the mesenchyme and the luminal airway epithelium. (Original magnification ×400, scale bar = 100 μm). There were no significant differences of protein localization. Epithelial and mesenchymal immunoreactivity for Hdac1 was stronger in EA-TEF lungs than in the control group at the canalicular stage (E18). **(E–H)** Representative immunoblotting and densitometric analysis of Hdac1 **(E,F)** and H3K56ac **(G,H)** protein in fetal lungs at E18. Results were normalized against expression of β-actin or total H3 respectively. ^*^P < 0.05, vs. age-matched controls.

The epigenetic mechanisms underlying the changes of gene expression in lung tissue were investigated by assaying the transcription of DNA methyltransferase (Dnmt)1 and histone deacetylase (Hdac) genes. The specificity of primers for epigenetic regulatory factors was confirmed by agarose gels electrophoresis (Figure 6E). Quantitative PCR did not find differences of *Dnmt1, Hdac2*, and *Hdac5* mRNA expression in normal and EA-TEF lungs (Figures 6A–D). *Dnmt1* mRNA expression in both groups was increased at E18. *Hdac1, Hdac2*, and *Hdac5* expression remained relatively constant in control lungs at all three developmental stages and was similar in both groups except that *Hdac1* mRNA was significantly upregulated in hypoplastic lungs compared with the control lungs at E18. Positive immunoreactivity for Hdac1 was seen in mesenchymal cells, in vascular structures in the mesenchyme, and in the luminal airway epithelium during the canalicular and saccular stages. There were no significant differences concerning protein localization, but epithelial and mesenchymal immunoreactivity were stronger in the EA-TEF model than in the control group at the canalicular stage (E18) (Figures 7A,B) but it restored to normal level at E21 (Figures 7C,D). Comparable changes in Hdac1 protein concentrations were observed in the immunoblotting assays (Figures 7E,F). Hdac1 regulates the acetylation state of histone H3 Lys56 (H3K56), and H3K56ac level was significantly decreased in EA lungs (Figures 7G,H).

## Discussion

Persistent nonspecific respiratory symptoms are commonly presented in children with EA, including brassy cough, bronchitis, pneumonia, asthma-like symptoms, and bronchial hyperresponsiveness [19–23]. Developmental abnormalities of tracheal structure and innervation or lung hypoplasia may account for sequelae that persist into childhood [3, 7, 24, 25]. We previously reported significant underdevelopment of the lungs in experimental EA because of defective embryonic airway branching. These findings support investigation of the mechanisms that underlie defective lung development in EA-TEF patients.

The genetic program of fetal lung development is a complex, tightly regulated process that is influenced by epigenetic and environmental factors during embryogenesis. Ohuchi et al. demonstrated that Fgf10 signaling played a key functional role in early embryonic lung epithelial and endothelial crosstalk during branching morphogenesis [13]. Fgf10 produced by lung mesenchymal cells acts as a chemotactic factor on the adjacent epithelium, and temporal and spatial expression of Fgf-10 determine the patterning of lung epithelial tubes. Fgf10 knockout mice exhibit complete lung agenesis, which illustrate the key role played by of Fgf10 during lung development [26]. Decreased expression of Fgf10 was reported in rodents nitrofen-induced pulmonary hypoplasia, furthermore exogenous Fgf-10 during fetal lung development in mice increased branching of nitrofen-exposed lungs [27]. FGF-10 also reduced in the lung tissue of infants with bronchopulmonary dysplasia [28]. Expression of Bmp4 and Ctsh are induced by Fgf10 in the distal lung epithelium during branching [29]. Shh, a negative regulator of Fgf-10 expression, is expressed throughout the epithelium of the developing lung, but most strongly in the distal epithelial tips [30]. This study investigated epithelial Fgf10 receptors and other pathway components during lung development in experimental EA-TEF induced by doxorubicin. Consistent with lung underdevelopment, Bmp4 and Ctsh expression were downregulated during the saccular stage. We speculated that decreased Fgf10 expression might have led to the reduction of Bmp4 and Ctsh associated with inhibition of the branching of lung epithelium. However, *Shh* expression was unchanged in the model embryos, indicating that other distal epithelial signals regulated mesenchymal Fgf10 expression.

Fgf10 selectively regulates differentiation of the cell lineages in both the proximal conducting airways and the distal respiratory airways. The alveolar epithelium contains type I and type II alveolar cells; the bronchial epithelium includes of Clara cells, ciliated cells, and neuroendocrine cells [31]. Sftpc is synthesized only in, and is a specific marker of type II cells [32], as other surfactant proteins including Sftpa and Sftpb are also synthesized in Clara cells. These surfactants are indispensable for the maintenance of alveoli and host defense [33]. Clara cell secretory protein (Ccsp; also known as Scgb1a1, Cc10, and uteroglobin) is secreted by Clara cells into the mucus that covers the bronchial epithelium of the mammalian lung. Because it is abundant in the airway mucus [34], Scgb1a1 has been proposed as a marker of airway epithelial damage [35]. Studies in Scgb1a1-deficient mice have indicated that Scgb1a1 is an endogenous inhibitor of Pla2 in pulmonary inflammation [18, 36]. In this study, Sftpc and Scgb1a1 expression were downregulated in the lungs of doxorubicin treated fetus, suggesting impaired development of lung epithelium as a cause of persistent respiratory symptoms.

Epigenetic regulation of gene expression is important for integrating signaling input and transcriptional output during fetal lung development [37]. HDACs regulate chromatin compaction, and aberrant HDAC expression and activity leads to defective lung development, and has been associated with the severity of COPD disease [38, 39] Currently, little is known about how doxorubicin affects the epigenetic control of embryonic lung development in EA models. In this study, enhanced Hdac1 expression in hypoplastic lungs may have resulted in deacetylation of lysine 56 of H3. Decreased H3K56ac levels have been positively correlated with changes in gene expression [40] might result in downregulated expression of epithelial proteins required for normal branching in EA models. Chromatin immunoprecipitation and quantitative PCR would be useful in investigating the contribution of histone modification at specific gene promoters to regulation of gene expression in experimental AE models.

In summary, disturbed molecular signaling in lung morphogenesis involving Fgf10/Ctsh resulted in impaired airway branching and consequent impairment of epithelial cell development, as evidenced by decreased Sftpc and Scgb1a1 expression. Recently, Xin Sun et al reported that Fgf-activated transcription factors, Etv4 and Etv5 control Fgf-Shh feedback loop in lung branching. Possible changes of ETV transcription factor in EA-TEF lungs and its effects on lung development deserved further investigation [41]. Upregulated Hdac1 and decreased H3K56ac may have been responsible for decreased expression of epithelial proteins. The findings invite support further study of epigenetic regulation and correction of the defective lung development in EA-TEF patients.

## Author contributions

XL conceived and designed the experiments, and wrote the manuscript. JW and HL performed the biological experiments and analyzed the data. LG managed the animals.

### Conflict of interest statement

The authors declare that the research was conducted in the absence of any commercial or financial relationships that could be construed as a potential conflict of interest.

## Supplementary material

Figure S1 Esophageal and pulmonary anatomy of esophageal atresia rat.
