# Supplementary figures and images for: Impaired FGF10 Signaling and Epithelial Development in Experimental Lung Hypoplasia With Esophageal Atresia
Source: Front Pediatr. 2018 Apr 20;6:109. doi: 10.3389/fped.2018.00109 (PMC5921531; doi:10.3389/fped.2018.00109)

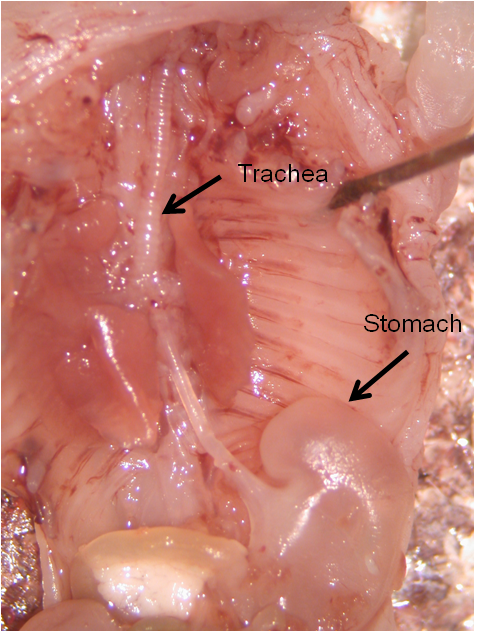

Supplement: Figure S1 — Esophageal and pulmonary anatomy of esophageal atresia rat. [file Image1.tiff]
